# Mobilization of IMEs Integrated in the *oriT* of ICEs Involves Their Own Relaxase Belonging to the Rep-Trans Family of Proteins

**DOI:** 10.3390/genes11091004

**Published:** 2020-08-26

**Authors:** Virginie Libante, Nazim Sarica, Abbas Mohamad Ali, Chloé Gapp, Anissa Oussalah, Gérard Guédon, Nathalie Leblond-Bourget, Sophie Payot

**Affiliations:** Université de Lorraine, INRAE, DynAMic, F-54000 Nancy, France; virginie.libante@univ-lorraine.fr (V.L.); sarica_n@hotmail.fr (N.S.); abbas.mohamadaLi94@gmail.com (A.M.A.); chloe.gapp3@etu.univ-lorraine.fr (C.G.); anissa.oussalah5@etu.univ-lorraine.fr (A.O.); gerard.guedon@univ-lorraine.fr (G.G.); nathalie.leblond@univ-lorraine.fr (N.L.-B.)

**Keywords:** integrative mobilizable elements, integrative conjugative elements, excision, gene transfer, conjugation, mobilization, origin of transfer, relaxase, MobT, antibiotic resistance, *lsa*(C)

## Abstract

Integrative mobilizable elements (IMEs) are widespread but very poorly studied integrated elements that can excise and hijack the transfer apparatus of co-resident conjugative elements to promote their own spreading. Sixty-four putative IMEs, harboring closely related mobilization and recombination modules, were found in 14 *Streptococcus* species and in *Staphylococcus aureus*. Fifty-three are integrated into the origin of transfer (*oriT*) of a host integrative conjugative element (ICE), encoding a MobT relaxase and belonging to three distant families: ICE*St3*, Tn*916*, and ICE*6013*. The others are integrated into an unrelated IME or in chromosomal sites. After labeling by an antibiotic resistance gene, the conjugative transfer of one of these IMEs (named IME_*oriTs*) and its host ICE was measured. Although the IME is integrated in an ICE, it does not transfer as a part of the host ICE (no *cis*-mobilization). The IME excises and transfers separately from the ICE (without impacting its transfer rate) using its own relaxase, distantly related to all known MobT relaxases, and integrates in the *oriT* of the ICE after transfer. Overall, IME_*oriTs* use MobT-encoding ICEs both as hosts and as helpers for conjugative transfer. As half of them carry *lsa*(C), they actively participate in the dissemination of lincosamide–streptogramin A–pleuromutilin resistance among Firmicutes.

## 1. Introduction

Integrative conjugative elements (ICEs) are mobile genetic elements (MGEs) integrated in bacterial chromosomes (or plasmids) that are able to excise as a circular form and transfer autonomously by conjugation (see [1,2] for reviews). These elements are widespread in bacterial genomes, even more than conjugative plasmids in almost all bacterial clades [3]. Therefore, they probably play a key role in horizontal transfer of genes, in particular, in virulence and antibiotic resistance spread [4,5]. ICE transfer is thought to be similar to transfer mediated by conjugative plasmids. The mechanistic aspects of conjugative transfer of plasmids are quite well characterized in Gram-negative bacteria and, more particularly, Proteobacteria, but conjugative transfer of ICE is less well known. Initiation of conjugative transfer requires nicking of DNA at a sequence called origin of transfer (*oriT*) by a protein called relaxase (or Mob for mobilization protein). The relaxase binds covalently to DNA and, by interacting with a so-called coupling protein (CP), brings one of the strands of DNA to an active transport system encoded by the conjugative element. This system of mating pair formation (MPF) is a macromolecular protein complex belonging to the type IV secretion systems (T4SS) [6]. The relaxase initiates not only the transfer but also intercellular rolling-circle replication (RCR), one strand being replicated in the donor strain, and the other strand being transferred in the recipient cell. In Gram-negative bacteria, relaxases belong to seven unrelated or very distantly related families that differ by their catalytic domains [3,6,7]. It should be emphasized that initiators of intercellular RCR, which are involved in the maintenance of plasmids, are unrelated to all families of relaxases from Gram-negative bacteria. All CPs from these bacteria are related and belong to the VirD4 family while MPFs were divided in six distantly related families [3,8]. The mechanisms of conjugation remain poorly understood in other clades, such as Firmicutes, a major group of Gram-positive bacteria, although they most often seem to be similar to those of Proteobacteria [6,9]. The known relaxases from Firmicutes belong to four of the six canonical families found in Proteobacteria and to two novel ones: MobM and MobT [7]. MobM relaxases belong to the tyrosine recombinase family and MobT relaxases belong to the Rep_trans family (with a conserved domain: PF02486) of RCR initiators. MobM relaxases are only encoded by plasmids belonging to the pCW3 family and MobT relaxases are only encoded by a large superfamily of widespread ICEs (Tn*916* superfamily), which includes distantly related families such as Tn*916*, ICE*St3*, and ICE*6013* families. All MobM-encoding plasmids and all MobT-encoding ICEs encode TcpA CPs that are unrelated to VirD4 [8] and MPFs belonging to a novel family, MPF_FATA_ [10].

Another class of integrated mobile genetic elements, the integrative mobilizable elements (IMEs), are able to transfer by conjugation although they do not encode the MPF. All of the very few exhaustive searches of IMEs and ICEs in genomes revealed more IMEs than ICEs [11,12,13,14]. However, IMEs are difficult to detect and therefore remain very poorly studied [1,11]. Like ICEs, they can contribute to the fitness of their bacterial host by bringing new traits such as antibiotic resistance [11,12]. Like ICEs, they use a tyrosine recombinase, a serine recombinase, or a DDE transposase to excise from and integrate into the chromosome or plasmids. Unlike ICEs, they hijack or subvert the DNA transport system encoded by unrelated conjugative elements (ICEs or plasmids) to promote their own transfer [1,11]. To be mobilized by ICEs or plasmids, they need to harbor their own origin of transfer. Some do not encode a relaxase, so they carry an *oriT* that is sufficiently similar to that of their helper conjugative element to be recognized by its relaxase. Almost all IMEs from Firmicutes characterized until now encode their own relaxase that always belongs to the canonical families also found in Proteobacteria. However, most of the 144 putative IMEs revealed by the analysis of 124 genomes of streptococci encode putative relaxases belonging to five families of RCR initiators of viruses or plasmids [13]. Many of the IMEs that encode a putative relaxase belonging to RCR initiators encode a putative CP related to TcpA CPs [11]. Some of the 144 putative IMEs found by Coluzzi et al. [13] are integrated inside a host ICE, resulting in a matryoshka element. They encode a putative relaxase suggesting that they are mobilized *in trans* by conjugative elements. However, their integration in ICEs suggests that they could also be transferred as a part of the ICE (mobilization *in cis*, as defined previously [1]). All of these target genes are very conserved or essential to ICE transfer (for example, the gene encoding the VirD4 CP of the ICE) [12,13].

We previously identified a family of putative IMEs that are integrated inside the *oriT* of ICEs belonging to the Tn*916* superfamily (called IME_*oriTs*) [15]. Such IMEs were detected in four strains of *Streptococcus agalactiae*, in two strains of *Streptococcus mutans*, in one strain of *Streptococcus mitis*, and in one strain of unidentified *Streptococcus* species. Three of them carry a *lsa*(C) gene, conferring cross-resistance to lincosamides, streptogramin A, and pleuromutilins in *S. agalactiae* [16]. The insertion of the IME in a sequence of *oriT*, which is conserved in diverse and broadly distributed ICEs from Firmicutes, provides a safe and widespread site of integration. Douarre et al. [17] later identified four additional IMEs of the IME_*oriT* family by examining 531 genomes of *S. agalactiae*. They performed conjugation experiments in order to study the transferability of two IMEs and of their cargo *lsa*(C). Despite repetitive experiments, only one transconjugant was obtained. Characterization of this transconjugant indicated that the IME_*oriT* was present in two copies (one integrated in a resident Tn*916* and another in a resident ICE belonging to the ICE*St3* family that is specifically integrated in a tRNALys gene). Transfer of the ICE hosting IME_*oriT* (specifically integrated in *rpsI*) in the donor strain was not observed [17]. This raises interesting evolutionary questions about the interactions between IMEs and their helper and/or host conjugative element. If IME integration disrupts an essential sequence or gene of the ICE, does it impair ICE transfer or does the IME excise when the transfer system of the ICE is activated (as previously suggested by Coluzzi et al. [13])? Is the IME only mobilized *in trans*, thus enabling its co-transfer with the ICE? Does the use of the ICE T4SS by the IME interfere with ICE transfer? Overall, is this IME a harmful pirate or a parasite of the ICE that hampers ICE transfer or a gentle hitchhiker that pacifically co-exists with the ICE?

In this study, an exhaustive search of this family of IMEs that target *oriT* of ICEs revealed 64 elements, not only in many species of streptococci but also in one strain of *Staphylococcus aureus*. In order to answer the questions raised above, we studied excision and transfer by conjugation of an IME_*oriT*. For these purposes, we made genetic constructs to create adequate donor and recipient strains that were then used in various conjugation experiments. These mating assays demonstrated that this IME_*oriT* can be mobilized *in trans* but not *in cis* by the ICE, and does not interfere with ICE transfer.

## 2. Materials and Methods 

### 2.1. In Silico Search and Analysis of IMEs Encoding a Relaxase or an Integrase Related to the One of IME_SagLMG15084_oriT 

The relaxase (GenBank ID: EPW57073.1) and integrase (GenBank ID: EPW57075.1) of IME_*SagLMG15084_oriT* described previously [15] were used as queries to find homologs by BlastP and tBlastN analyses (with default parameters on non-redundant protein sequences and nucleotide databases, respectively; last NCBI database interrogation on 2020/02/11). Hits with more than 40% of amino acid identity with the whole query sequence were further analyzed. Sequences adjacent to the identified genes were extracted from genomes or contigs and then manually explored using Geneious Prime (version 2020.0.5, Biomatters Ltd., Auckland, New Zealand to identify the boundaries of the elements (search of direct repeats). IME_*oriTs* were also identified using a previously described method [13] on 21 genomes of *Streptococcus salivarius* sequenced in our laboratory (see more details in the accompanying paper [14]) and were added to the set of IMEs. Denominations of the IMEs include letters for the species (Sag for *S. agalactiae*, San for *Streptococcus anginosus*, Saur for *S. aureus*, Saus for *Streptococcus australis*, Sco for *Streptococcus constellatus*, Smi for *S. mitis*, Smu for *S. mutans*, Sora for *Streptococcus oralis*, Spn for *Streptococcus pneumoniae*, Spse for *Streptococcus pseudopneumoniae*, Ssal for *S. salivarius*, Ssan for *Streptococcus sanguinis*, Ssp for unspecified *Streptococcus* species, and Sth for *Streptococcus thermophilus*) and the name of the bacterial strain that hosts the IME. Denominations also include the name of the target sequence (*oriT* or oriT* if the IME is integrated in a secondary site). A multiple alignment of the IME sequences was made using Clustal Omega 1.2.2 in order to construct a full distance matrix. This enabled the selection of representative sequences for further analyses. Based on the distance matrix obtained for IME sequences, sequences were aligned two-by-two using BlastN (with default parameters). These comparison files were then used to draw alignment graphs using Artemis Comparison Tool (ACT) provided by the Sanger Centre [18]. Manual editing of the comparison figure was performed using Inkscape.

Cargo genes of the putative IMEs were analyzed using the Conserved Domain search tool on NCBI website (https://www.ncbi.nlm.nih.gov/Structure/cdd/wrpsb.cgi).

### 2.2. Construction of Phylogenetic Trees

Sequences of the integrase genes, relaxase proteins and Lsa(C) proteins encoded by the putative IMEs were extracted using Geneious Prime (version 2020.0.5, Biomatters Ltd.) and aligned using ClustalW. Phylogenetic trees were built with MEGA7 [19] using the maximum likelihood method based on the Tamura-Nei model [20] without outgroup. Initial tree(s) for the heuristic search were obtained automatically by applying neighbor-joining and BioNJ algorithms to a matrix of pairwise distances estimated using the maximum composite likelihood approach and then selecting the topology with superior log likelihood value.

### 2.3. Bacterial Strains and Growth Conditions

The bacterial strains used in this study are indicated in Table 1.

*S. salivarius* and *S. thermophilus* strains were grown in M17 broth supplemented with 0.5% lactose (LM17) at 37 or 42 °C, respectively, without shaking. Solid cultures were made in oxygen-free environment induced by GasPak utilization (BioMérieux, Marcy l’Etoile, France). When required, cultures were supplemented with the following antibiotics: chloramphenicol (4 µg·mL^−1^ for *S. thermophilus* or 8 µg·mL^−1^ for *S. salivarius*), erythromycin 5 µg·mL^−1^, spectinomycin (500 µg·mL^−1^ for *S. thermophilus* or 800 µg·mL^−1^ for *S. salivarius*), and kanamycin (500 µg·mL^−1^ for *S. thermophilus* or 800 µg·mL^−1^ for *S. salivarius*).

### 2.4. IME and ICE Tagging with a Resistance Gene and Deletion of Integrase and Relaxase Genes of the Integrative Elements

Overlap PCR was used to insert an antibiotic resistance gene in integrative elements of *S. salivarius* L25 as described previously [21]. Deletion of the integrase genes (of ICE *SsalF1-4_fda* in *S. salivarius* F1-4 and ICE*St3* of *S. thermophilus* in LMG18311) was done by insertion of a chloramphenicol resistance cassette as described previously [24]. IME_*SsalL25_oriT* and 1-kb-upstream and downstream flanking regions were PCR-amplified before natural transformation of *S. thermophilus* LMG18311(ICE*St3*) as described by Gardan et al. [25]. The deletion strategy, used to construct IME_*SsalL25_oriT*Δ*rel* to avoid introducing an antibiotic resistance gene and polar effect, was similar to the strategy used previously [23]. Sequence of the primers used is indicated in Table S1. 

### 2.5. Excision Tests and Mating Experiments

Excision tests were done as described previously [21]. Sequence of the primers used is indicated in Table S1. Relevant amplicons, *attI* and *attB* were sequenced (Eurogentec, Angers, France).

Mating experiments using *S. salivarius* and *S. thermophilus* strains were made as described previously [21,24]. All the experiments done are listed in Table S3. At least three technical replicates of three independent biological replicates were done. Data are expressed as means ± SD. Statistical analysis was performed using the paired Students *t*-test. A *p*-value < 0.05 was considered as significant.

## 3. Results

### 3.1. Integrative Mobilizable Elements (IMEs) That Target oriT of Other Integrative Elements (IME_oriTs) Are Widespread in Firmicutes and Carry Different Cargo Genes

#### 3.1.1. Distribution of IME_*oriTs* in Firmicutes

All the IMEs integrated in an *oriT* of an ICE that were previously described [15,17] encode very closely related putative relaxases and integrases (92 to 100% of amino acid identity). We chose to use the sequence of the putative relaxase and the integrase of IME_*SagLMG15084_oriT* to search for homologs in bacterial genomes available on the NCBI website and in 21 genomes of *S. salivarius* sequenced in our laboratory [14]. Sixty-nine proteins very closely related (91 to 100% of amino acid identity) to the relaxase of IME_*SagLMG15084_oriT* were found in 14 different *Streptococcus* species and in one strain of *S. aureus.* Analysis of these putative relaxases indicate that they all exhibit a PF2486 domain characteristic of Rep_trans_proteins. This family includes the MobT relaxases of ICEs belonging to the Tn*916* superfamily (Tn*916*, ICE*Bs1*, ICE*St3*) and the RCR initiators involved in the maintenance of many small plasmids of Firmicutes (for example, plasmids of the pT181 family like RepC of pT181 and RepD of pC221 [26]). Even though the three groups of Rep-trans proteins are only very distantly related (less than 20% of amino acid identity except for RepSTK1, see the phylogenetic tree presented in Supplementary Figure S3), sequence alignment indicate that they all display the five previously described characteristic motifs that include three acidic residues involved in cation coordination and a catalytic tyrosine [23,26]. Sequences adjacent to the putative relaxase and integrase genes were analyzed in order to identify and delineate putative IMEs. All the relaxase genes were found associated with direct repeats and an integrase gene, two characteristics of integrative elements. In five of these elements (elements found in two strains of *S. thermophilus*, in two strains of *S. anginosus*, and in one strain of *Streptococcus equinus*), the integrase gene is truncated (pseudogene). Thus, these elements were considered as defective and were not included in Table S2 that synthetizes results of the in silico search. Among the 64 detected putative IMEs, several almost identical elements were found in various species (Table S2): (i) IME_*SagGB00984_oriT* in 10 other strains of *S. agalactiae*, one strain of *Streptococcus cristatus* (98% of nucleotide identity) and one strain of *S. mitis* (99% of nucleotide identity); (ii) IME_*SagUCN70* carrying *lsa*(C) found in another strain of *S. agalactiae*, four strains of *S. salivarius*, four strains of *Streptococcus parasanguinis*, two strains of *S. pseudopneumoniae*, and two strains of *Streptococcus* sp. (98 to 99% of nucleotide identity), and (iii) IME_*SaurER01570.3_oriT* carrying *lsa*(C) found in one strain of *S. aureus*, one strain of *S. oralis*, and two strains of *Streptococcus* sp. (100% of nucleotide identity).

#### 3.1.2. Chromosomal Integration Sites of IME_*oriTs*

In most of the cases (*n* = 53), integration of the IME occurred upstream the gene encoding the MobT relaxase: in Tn*916*-related ICEs (16 ICEs closely related to Tn*916* that integrate in low G+C regions, one Tn*5801* integrated in the 3′ end of *guaA*), in ICE*St3*-related ICEs (15 integrated in the 3′ end of a tRNALys gene, six in the 3′ end of *fda*, 7 in the 3′ end of *rpsI*, four in the 5′ end of a *ebfC* and three in the 3′ end of *rpmG*), and in one ICE related to ICE*6013* (that integrates at many different sites [27]) in *S. aureus* (Table S2). For ICEs of the Tn*916* superfamily, this region includes the origin of transfer (*oriT*) of the ICE [23,28]. As described previously [15], the IME more precisely integrates in a stem–loop sequence that likely includes the nicking site of the relaxase (by homology with the *nic* sequence demonstrated for ICE*Bs1* by Lee and Grossman [29]). Interestingly, an IME_*oriT* was also detected inside an unrelated IME (integrated in *rpmG*) between the gene encoding a putative relaxase (that belongs to the Rep_trans family, is devoid of HTH domain and is distantly related to the known MobT relaxases) and the gene encoding its putative TcpA CP (Table S2). In addition, eight of these putative IMEs were found integrated elsewhere in the chromosome (not inside another MGE). The integrases of these eight IMEs do not cluster together on a phylogenetic tree (see sequences indicated by a star in Figure S1). In the same way, the integrases of IMEs targeting different subfamilies of ICEs (Tn*916*-related elements, ICE*St3* elements or ICE*6013*) or different integration sites (in particular those targeting *fda*, *rpsI*, or *rpmG*) do not group together on the phylogenetic tree (Figure S1). Elements with identical integrases were even found integrated in different subfamilies of ICEs. 

#### 3.1.3. Cargo Genes of IME_*oriTs*

**Figure 1:**
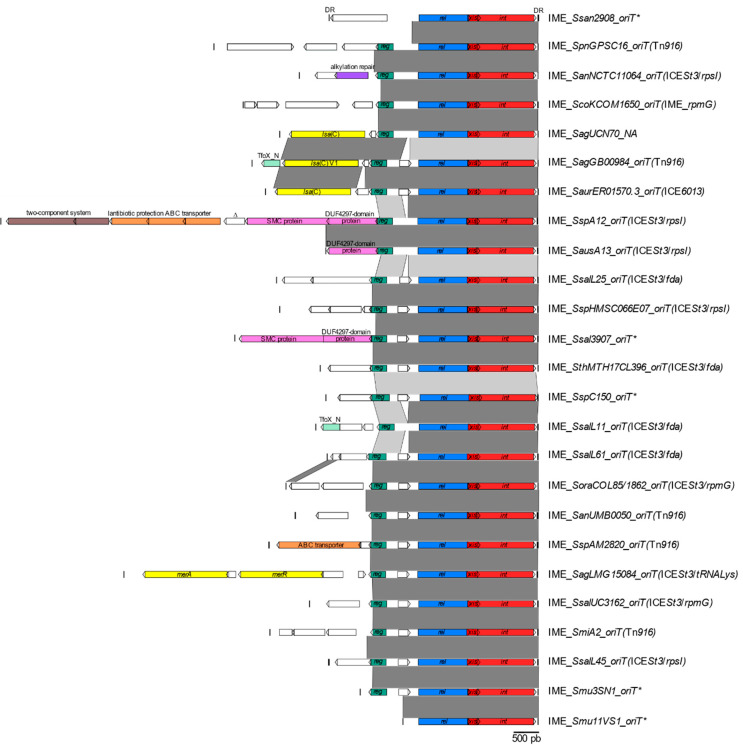
Diversity of the IMEs carrying a relaxase related to the one of IME_*SagLMG15084_oriT*. IMEs exhibiting more than 85% of nucleic acid identity on the whole sequence with another one are not indicated on the figure (see Table S2 for more details). The bacterial host species (Sag for *Streptococcus agalactiae*, San for *Streptococcus anginosus*, Saur for *Staphylococcus aureus*, Saus for *Streptococcus australis*, Sco for *Streptococcus constellatus*, Smi for *Streptococcus mitis*, Smu for *Streptococcus mutans*, Sora for *Streptococcus oralis*, Spn for *Streptococcus pneumoniae*, Ssal for *S. salivarius*, Ssan for S*treptococcus sanguinis*, Ssp for unspecified *Streptococcus* species, and Sth for *S. thermophilus*) and the name of the strain is indicated in the name of the IME. When the IME is integrated in the origin of transfer of a mobile genetic element (MGE), the targeted ICE/IME is shown in brackets. oriT* is used for IMEs integrated elsewhere in the chromosome and NA when information is not available. Direct repeats (DRs) delimiting IMEs are shown as black bars. Coding DNA sequences (CDSs) appear as arrows (truncated genes are indicated by Δ). The recombination genes (encoding the integrase (*int*) and the recombination directionality factor (RDF) also known as excisionase (*xis*)) appear in red, the relaxase gene (*rel*) in blue, and the regulation gene (*reg*) in green. Genes encoding a hypothetical protein with unknown function appear in white. Cargo genes encoding proteins with a putative function inferred from an in silico analysis are indicated in color: in purple for a putative alkylation repair protein, in yellow for resistance operon/genes, in light green for a putative regulator (with a TfoX_N domain), in brown for a two-component system, in orange for ABC transporters, and in pink for a putative chromosome segregation protein (SMC) and its adjacent gene (encoding a protein with a DUF4297 domain). A nucleic acid sequence identity between sequences higher than 75% is indicated in light grey and in dark grey when higher than 90%.

In addition to the relaxase and recombination genes, required for the mobility of the IME and a regulator gene (found in all IME_*oriTs*, except two), all the IME_*oriTs* detected by the in silico analysis carry diverse cargo genes (Table S2 and Figure 1). The sole exception is IME_*Smu11VS1_oriT* that contains only the three mobility genes (Figure 1). This diversity of cargo genes has an impact on the length of the IMEs that expands from 2.7 to 10.9 kb (IME_*Smu11VS1* and IME_*SspA12_oriT*, respectively, Figure 1). 

Most of these cargo genes encode putative hypothetical proteins but some of them encode a protein with a specific domain that could give information about the possible function of the protein (Table S2).

Several encode proteins that could confer resistance against antibiotics, bacteriocins, or heavy metals (Table S2). In particular, half (*n* = 34) of the detected IMEs carry a *lsa*(C) resistance gene that is known to confer cross-resistance to lincosamides, streptogramin A, and pleuromutilins in *S. agalactiae* [16]. Douarre et al. [17] described in *S. agalactiae* a variant of Lsa(C) that shares 93% of amino acid identity with Lsa(C) found initially in *S. agalactiae* strain UCN70 [16]. This variant was found in 11 strains of *S. agalactiae* (including *S. agalactiae* GB00984, Figure 1), in one strain of *S. mitis*, and in one strain of *S. cristatus* (Table S2). These IMEs are integrated in different sites (inside a Tn*916-*related element, inside an ICE*St3* element integrated in a tRNALys gene, and in the other locations indicated as oriT*). A second variant of Lsa(C) showing 96% of amino acid identity with Lsa(C) of *S. agalactiae* strain UCN70 was discovered in two strains of *S. pseudopneumoniae* (Table S2). In addition, *Streptococcus* sp. AM28-20 could encode an ABC-type multidrug transport system. Strain A12 also likely encodes an ABC transporter that could confer protection against lantibiotics (proteins with TIGR03732, TIGR03733, and TIGR03740 domain, respectively, Table S2). Lastly, a heavy metal resistance gene (Hg^++^ resistance operon) was found on the IMEs hosted by *S. agalactiae* LMG15084, 18RS21, and NCTC11079.

In silico analysis indicated that IME_*oriTs* also convey genes that are not involved in resistance: a putative alkylation repair protein encoded by an IME found in *S. anginosus* (strain NCTC11064), a putative regulator (N terminal domain of TfoX competence regulator in 6 strains of *S. agalactiae*, *S. salivarius* L11, and *S. cristatus* BCA6), a two-component system (in strain A12), and a protein with a domain found in chromosomal segregation proteins (in strains A12 and *S. salivarius* 39-07) (Figure 1 and Table S2). 

### 3.2. IME_oriTs Identified in S. salivarius Strains L11, L25 and L45 Are Able to Excise from the Chromosome

**Figure 2:**
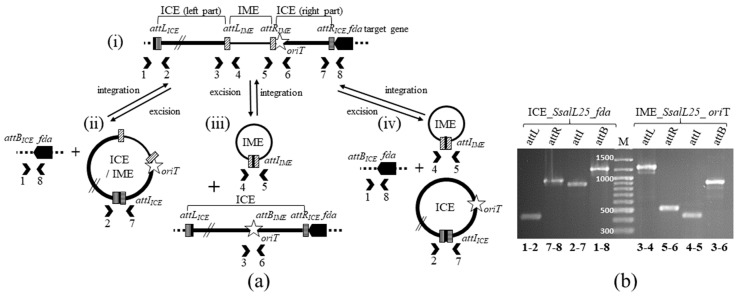
Excision of IME_*SsalL25_oriT* and its hosting ICE (ICE_*SsalL25_fda*) in *S. salivarius* L25. (**a**) Diagram showing the localization of PCR primers (numbered chevrons) used to detect (i) integrated forms of elements (*attR* and *attL* for right and left junctions, respectively) and excised forms (*attI* for circular form of the integrative elements and *attB* for the bacterial empty site) if (ii) the ICE/IME composite element excises, (iii) only the IME excises or (iv) both elements excise separately; (**b**) PCR products obtained with the primer pairs (numbers indicated at the bottom of the gel) when analyzing excision of IME_*SsalL25_oriT* and of its hosting ICE (ICE_*SsalL25_fda*). The sizes of the PCR fragments were confirmed by parallel migration of a DNA ladder (M for marker on the figure). The primers pairs used for these amplifications and expected sequence lengths are listed in Table S1.

In both L11 and L25 *S. salivarius* strains, an IME_*oriT* is integrated into *oriT* of an ICE of the ICE*St3* family, which is integrated in the 3′ end of *fda*. In *S. salivarius* L45, another IME_*oriT* is integrated in *oriT* of an ICE*St3*-related ICE integrated in the 3′ end of *rpsI*. Excision of these three IMEs and of their host ICEs was tested by PCR for wild type strains. All the six integrative elements (IMEs and ICEs) were found to excise (Figure 2, data shown for elements of strain L25).

### 3.3. IME_oriT of S. salivarius Strain L25 (IME_SsalL25_oriT) Can Be Transferred by Conjugation to Another S. salivarius Strain and to S. thermophilus

**Figure 3:**
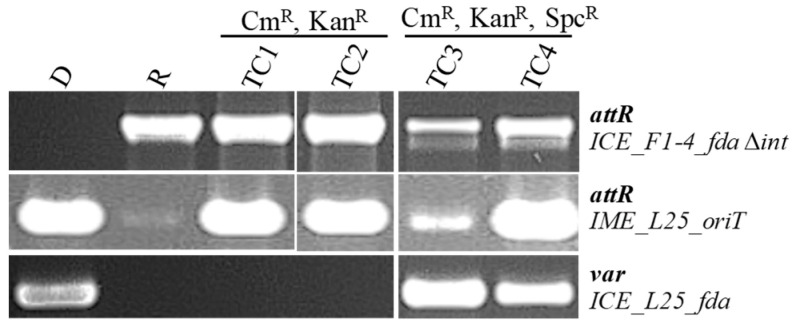
PCR analysis of four transconjugants (TC1 to TC4) obtained using the mating pair *S. salivarius* L25(ICE_*SsalL25_fda*::IME_*SsalL25_oriT*) (donor D)/*S. salivarius* F1-4(ICE_*SsalF1-4_fda*Δ*int*) (recipient R). Specific regions were amplified to discriminate the elements *attR* for ICE_*SsalF1-4_fda*Δ*int* and IME_*SsalL25_oriT* and the region of ICE_*SsalL25_fda* that includes accessory genes (different from the one of ICE_*SsalF1-4_fda*), indicated as *var* in the figure. Resistance phenotypes of the transconjugants are indicated above the wells: Cm^R^, chloramphenicol resistance (conferred by ICE_ *SsalF1-4_fda*Δ*int*); Kan^R^, kanamycin resistance (conferred by IME_*SsalL35_oriT*); and Spc^R^, spectinomycin resistance (conferred by ICE_*SsalL25_fda*).

IME_*SsalL25_oriT* and the ICE hosting this IME (ICE_*SsalL25_fda*) were labeled by kanamycin and spectinomycin resistance genes, respectively, to study their transfer by conjugation. The recipient strain used in the mating pair was *S. salivarius* strain F1-4 that hosts an ICE of the ICE*St3* family integrated in *fda*. To immobilize this ICE in the recipient strain, the integrase gene of this ICE was deleted and replaced by a chloramphenicol resistance gene (ICE_*SsalF1-4_fda*Δ*int*, Table 1). No excision of this ICE was detected (data not shown). Transfer was observed for both ICE_*SsalL25_fda* and IME_*SsalL25_oriT* (Figure 3). When transconjugants (TC) harbor the three elements, the sequences of *oriT*s of the two ICEs are too similar to determine whether the IME_*SsalL25*_*oriT* has a preferred insertion site. Some of these transconjugants carried only IME_*SsalL25_oriT* and ICE_*SsalF1-4_fda*Δ*int* (Figure 3). This does not exclude a co-transfer of ICE_*SsalL25_fda* in these transconjugants since the helper ICE could have been lost in the recipient strain after transfer due to the occupation of the *fda* site by ICE_*SsalF1-4_fda*Δ*int* in the recipient strain. 

Only a few transconjugants were obtained using a *S. salivarius* strain as recipient. This is in agreement with previous results indicating poor conjugative transfer in the *S. salivarius* species in laboratory conditions [21]. IME_*SsalL25_oriT* was thus transferred (by transformation) to a *S. thermophilus* strain (LMG18311) that proved proficient for ICE transfer [22,30] and carries ICE*St3* (labeled by a chloramphenicol resistance gene) [22]. ICE and IME present in this LMG18311 strain are able to excise (Figure S2). Further conjugation experiments were performed using this strain (LMG18311(ICE*St3*::IME_*SsalL25_oriT*)) as donor in the mating pairs. To increase the transfer frequency, we used *S. thermophilus* LMG18311Δ*epsE* mutant, as recipient strain [24]. Using the mating pair LMG18311(ICE*St3*::IME_*SsalL25_oriT*) (donor)/LMG18311Δ*epsE* (recipient), the frequencies of transfer of the ICE and of the IME were 3.3 × 10^−4^ ± 1.3 × 10^−4^ and 3.1 × 10^−6^ ± 1.5 × 10^−6^ TC/donor cell, respectively (Table S3). IME_*SsalL25_oriT* transfer was thus observed at a 100-fold lower frequency than ICE transfer. When the recipient strain already carries an ICE*St3* element (that offers an integration site for the IME*_oriT*) the frequency of TC obtention was 3-fold higher (9.3 × 10^−6^ ± 6.5 × 10^−6^ TC/donor cell) (Table S3).

### 3.4. IME_SsalL25_oriT Can Be Mobilized in Trans

**Figure 4:**
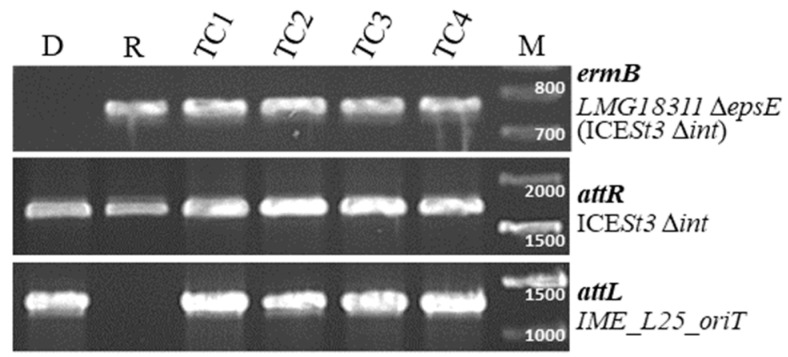
PCR analysis of four transconjugants (TC1 to TC4) obtained using the mating pair LMG18311(ICE*St3*Δ*int*::IME_*SsalL25_oriT*) (donor D)/LMG18311Δ*epsE* (ICE*St3*Δ*int*) (recipient R). Specific regions were amplified to discriminate the recipient strain, *ermB* for Δ*epsE*, and *attL* for IME_*SsalL25_oriT*. The integrase deletion (done by insertion of a chloramphenicol resistance cassette) was detected with the amplification of the *attR* site of ICE*St3*. The sizes of the PCR fragments were confirmed by parallel migration of a DNA ladder (M for marker on the figure). The primers pairs used for these amplifications and expected sequence lengths are listed in Table S1.

To check if IME_*SsalL25_oriT* can be mobilized *in trans*, other strains were constructed. The integrase gene of the ICE hosted by strain LMG18311(ICE*St3*) was deleted, resulting in LMG18311(ICE*St3*Δ*int*). Excision and conjugative transfer of this element were not detected. IME_*SsalL25_oriT* was transferred (by conjugation) in this strain to obtain LMG18311(ICE*St3*Δ*int::*IME_*SsalL25_oriT*). Then, *epsE* was also deleted in the LMG18311(ICE*St3*Δ*int*) strain to obtain LMG18311Δ*epsE(*ICE*St3*Δ*int*) strain. In conjugation experiments using the mating pair LMG18311(ICE*St3*Δ*int::*IME_*SsalL25_oriT*) as donor and LMG18311Δ*epsE*(ICE*St3*Δ*int*) as recipient, transconjugants were obtained at a frequency of 4.8 × 10^−6^ ± 4.0 × 10^−6^ TC/donor cell (Table S3). In each experiment, four transconjugants were analyzed by PCR and all of them exhibited the same profile (Figure 4). Since the ICE hosting the IME was immobilized in the donor strain, this indicated that IME_ *SsalL25*_*oriT* was mobilized *in trans*, i.e., excised and hijacked the conjugation machinery of the ICE to transfer by conjugation. A control conjugation experiment using the mating pair LMG18311(ICE*St3*Δ*int::*IME_*SsalL25_oriT*) (donor)/LMG18311Δ*epsE* (recipient without integration site for the IME) gave no transconjugant, indicating that the IME_ *SsalL25*_*oriT* integrates efficiently only in an *oriT* (Table S3).

### 3.5. The Relaxase of IME_SsalL25_oriT Is Needed for IME Mobilization and Does Not Contribute to the Transfer of the Helper ICE

The IME_*oriTs* encode a protein belonging to the Rep_trans family that is very distantly related to known MobT relaxases such as the one of ICE*St3* [23] and could be the relaxase of the IME. The gene of IME_*SsalL25_oriT* encoding this Rep_trans protein was deleted in strain LMG18311(ICE*St3*::IME_*SsalL25_oriT*) and conjugation experiments were performed using this strain (LMG18311(ICE*St3*::IME_*SsalL25_oriT*Δ*rel*)) as donor and the LMG18311Δ*epsE* (ICE*St3*Δ*int*) strain as recipient. No transfer of the IME was observed using this mating pair (Table S3). Thus, this protein is essential to its mobilization and cannot be complemented by the relaxase of the ICE. Therefore, it is the relaxase of the IME. Additional conjugation experiments were performed using the LMG18311Δ*epsE* strain as recipient. Despite a frequency of ICE transfer reaching 3.5 × 10^−4^ ± 2.0 × 10^−4^ TC/donor cell, no co-transfer of the IME was observed (Table S3). Therefore, no *cis*-mobilization (i.e., passive transfer as a passenger of the ICE) of IME_*oriT* could be detected.

IME_*SsalL25_oriT* was transferred by conjugation into a strain deleted for the relaxase gene of ICE*St3* to obtain LMG18311(ICE*St3*Δ*rel*::IME*_SsalL25*_*oriT*) used as donor in conjugation experiments with LMG18311Δ*epsE* strain as recipient. This ICE was still able to excise (data not shown) but no transconjugant was obtained (Table S3). Therefore, the relaxase of the ICE cannot be complemented (or at a very low efficiency that was undetectable in the laboratory conditions used) by that of the IME.

### 3.6. IME_SsalL25_oriT and Helper ICE Do Not Compete for the Conjugation Machinery

**Figure 5:**
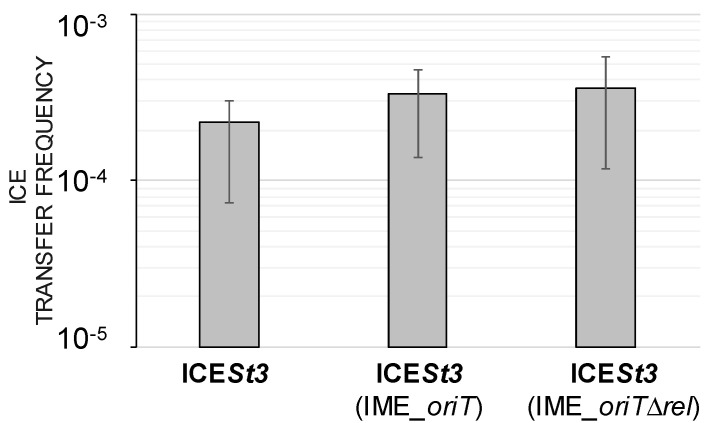
Frequencies of ICE*St3* transfer observed when the ICE does not host an IME_*SsalL25*_*oriT* or hosts a wild-type IME_ *SsalL25*_*oriT* or an IME deleted for its relaxase gene (IME_ *SsalL25*_*oriT*Δ*rel*). Results were obtained from four independent biological replicates.

Since IME_*SsalL25_oriT* needs to use the conjugation machinery of its helper ICE, we wondered whether it is at the expense of the ICE transfer rate. Thus, ICE*St3* transfer was measured for three distinct structures: ICE*St3* devoid of IME_*SsalL25_oriT*, ICE*St3* hosting an IME_*SsalL25_oriT* and ICE*St3* hosting an IME deleted for its relaxase gene (and thus unable to transfer [23]). Frequencies of ICE*St3* transfer were similar (Table S3). This indicates that, in these laboratory mating assay conditions, IME mobilization does not impair the ICE transfer rate (Figure 5).

Conversely, immobilization of the helper ICE in the donor cell (using Δ*int* mutant of ICE*St3*) does not have any impact on the IME transfer rate in the tested laboratory conditions (Table S3). This indicates that use of the conjugation machinery is not a limiting factor for ICE and IME transfer in the tested laboratory conditions.

## 4. Discussion and Conclusions

MobT relaxases from ICEs of the Tn*916* superfamily (Tn*916*, ICE*Bs1*, and ICE*St3*) belong to a larger family of proteins, Rep_trans, that are classically considered as RCR initiators mainly involved in plasmid maintenance [23,31]. None has ever been shown to be involved in the transfer of a mobilizable element. However, we recently found that many integrative elements from streptococci*,* which do not encode any MPF protein, encode Rep_trans “RCR initiators” that are very distantly related to genuine MobTs from ICEs [12,13,14,15]. We previously proposed that the “Rep-trans” proteins of these elements are MobT relaxases, enlarging this family, and that these elements are IMEs [13]. Here, we showed for the first time that (i) the Rep_trans “RCR initiators” encoded by one of these types of elements (IME_*oriTs*) are actually relaxases involved in their conjugative transfer; and (ii) these elements are IMEs that use the MPF_FA_ and TcpA of helper ICEs to promote their own transfer. This suggests that the tridimensional structures of the proteins are conserved despite a low conservation of the primary sequence, as previously described for the Rep proteins RepSTK1 of *Geobacillus stearothermophilus* and staphylococcal Rep proteins [26]. This works substantiates the very high prevalence of IMEs encoding relaxases related to RCR initiators involved in plasmid and virus replication [13]. It remains to be examined if plasmids harboring Rep-trans proteins can be mobilized by ICEs encoding a MobT relaxase. Such mobilization (by ICE*Bs1*) has been described for plasmids that encode RCR Initiators of the Rep_1 family [32]. In addition to their function in conjugative transfer, the MobT/Rep_Trans proteins of Tn*916* and ICE*Bs1* also catalyze the initiation of RCR needed for the maintenance of the excised ICEs [28,33]. As suggested previously, this could also be the case for the IMEs [11].

We previously described twelve members of this IME_*oriT* family [15]. In this study, the extensive search of related IMEs in available bacterial genomes led to the identification of 52 new IME_*oriT*. These 64 elements are distributed in 14 streptococcal species and in *S. aureus*. Almost identical elements were found in distant species, indicating a very recent horizontal transfer of these elements. These elements target the *oriT* of ICEs belonging to three distantly related families (Tn*916*, ICE*St3* and ICE*6013*) of the Tn*916* superfamily that all exhibit a MobT relaxase. More surprisingly, one IME_*oriT* was found inside an IME integrated in *rpmG*. It is worth mentioning that this IME also encodes a relaxase belonging to the enlarged MobT family. Therefore, this suggests that the IME_*oriT* recognizes specifically the *oriT* associated to MobT relaxases and more precisely a very short conserved sequence that includes the site that is nicked by the relaxase of a very large array of integrated elements encoding very distant relaxases. However, some of IME_*oriT* elements were also found integrated in other chromosomal sites (neither in ICEs nor in IMEs) even though they do harbor an integrase closely related to the one of the IMEs integrated in *oriT*. These sites could be secondary ones that present some similarities with the site targeted by the integrase. 

Diverse cargo genes were found on the IMEs of the IME_*oriT* family. Half of these elements carry *lsa*(C) that confers cross-resistance to lincosamides, streptogramin A, and pleuromutilins. Thus, these IMEs play a major role in the dissemination of this particular cross-resistance. Interestingly, one of these IMEs carrying *lsa*(C) is integrated in an ICE*6013*. This is only the second description of an ICE*6013* carrying an antibiotic resistance gene. The first description is an ICE*6013* carrying a penicillin resistance gene through insertion of Tn*552* but, in this case, the transposon disrupts the DDE transposase gene of the ICE and thus impairs its excision and transfer [27]. In the present example, the ICE likely remains active, provided that IME first excises. We recently reported the contribution of different families of IMEs to the dissemination of antibiotic resistance in *Streptococcus suis* [12]. IMEs have been very poorly studied until now because they are difficult to detect in bacterial genomes due to their small size and high diversity. However, their role in antibiotic resistance dissemination and, in particular, in streptococci, should be better considered. In addition, three IMEs carry *merA* that encodes a mercuric reductase conferring mercury resistance. This gene is frequently found on MGEs, in particular, in oral bacteria belonging to the Firmicutes group [34] due to the use of mercury in dental care in the past. Furthermore, one IME could encode an ABC transporter protecting its bacterial host from lantibiotics. Bacteriocins are ribosomally synthesized, antimicrobial substances produced by many bacteria to survive in competitive environments [35]. Bacteriocins from Firmicutes are classified into two main classes: (i) post-translationally modified bacteriocins (lantibiotics) and (ii) non-modified bacteriocins. Different mechanisms of immunity against lantibiotics have been described, including ABC transporters that export lantibiotics into the surrounding medium [36]. This family of IMEs could also contribute to the fitness of the host by providing an additional alkylation DNA repair protein. Alkylating agents, which are widespread in the environment, also occur endogenously in bacteria as primary and secondary metabolites. Such compounds have intrinsically extreme cytotoxic and frequent mutagenic effects, to which organisms have developed resistance by evolving multiple repair mechanisms to protect cellular DNA [37]. 

Although the elements are integrated in ICEs, the experimental characterization of the mobility of an IME_*oriT* failed to find any mobilization *in cis* by its host ICE. This suggests that disruption of the origin of transfer of the ICE by integration of the IME impairs ICE transfer and that prior excision of the IME is required to visualize ICE transfer. Furthermore, conjugative transfer of the IME by mobilization *in trans* was observed, albeit at a 100-fold lower frequency than that of ICE. This transfer does not impair the ICE transfer rate. This indicates that IME_*oriT* benefits from the DNA transport system encoded by their helper ICE but does not impair ICE transfer. Similar interactions could also occur between the many IMEs that integrate inside the genes encoding conserved or essential proteins (such as VirD4 CP) of ICEs of another superfamily widespread in Firmicutes (Tn*5252* superfamily) and their ICE host [11,12]. The impact of an IME on the transfer and/or stability of their helper co-resident element has been studied in depth only for IMEs of the SGI-1 family from the proteobacterium *Salmonella* [38,39,40,41,42]. Although these IMEs are mobilized only by conjugative plasmids of the IncA/C incompatibility group, SGI-elements and IncA/C plasmids are never found together in clinical multidrug resistant isolates. Firstly, the conjugative plasmids transfer from the donor cell to a recipient cell strain harboring the quiescent IME, inducing the excision of the IME. Secondly, the IME subverts the conjugation apparatus of the plasmid to invade the donor cells harboring IncA/C plasmids by retrotransfer and then probably invades all the cells of the population that harbor the plasmids. Thirdly, it suppresses plasmid transfer and leads to plasmid loss in the cell by interfering with plasmid regulation. Therefore, in contrast to the IME_*oriT* studied here, these IMEs are harmful pirates of conjugative IncA/C plasmids that await the arrival of the conjugative plasmid and then are probably transferred to all the cells harboring the plasmids and then eliminate these plasmids [41,42]. 

In conclusion, IMEs that integrate inside the origin of transfer of both ICEs and IMEs are widespread in streptococci and are also found in other Firmicutes. They vehicle cargo genes, particularly antibiotic resistance genes, and can thus participate in the dissemination of these threatening genes. They can also offer other helpful traits to their bacterial host (protection against bacteriocins or repair of alkylation damage), which help them persist in bacterial communities. The IME_*oriTs* are the first elements that are clearly shown to use a conjugative element both as a host for their integration and as helper supplying their transfer engine (CP and MPF). Some IMEs, like SGI1, can act as harmful pirates of their host/helper but some (such as IME_*oriTs*) can also be harmless hitchhikers of the ICEs. Other unrelated IMEs in Firmicutes may have similar capacities. Further studies are needed in order to decipher the mechanistic aspects of their mobilization.

## Figures and Tables

**Table 1:** Characteristics of the strains used in this study.

Bacterial Species	Strain Name (Name of the ICE*St3*-Related ICE ^1^, Which Hosts or Not an IME_*oriT* ^1^ Carried by the Strain)	Genotype ^1^	Reference
*Streptococcus salivarius*	L11(ICE_*SsalL11_fda* ::IME*_SsalL11*_*oriT*)	Wild type strain carrying ICE_*SsalL11_fda* hosting IME_ *SsalL11*_*oriT*	[14]
*S. salivarius*	L25(ICE_*SsalL25_fda* ::IME_*SsalL25*_*oriT*)	Strain carrying ICE_*SsalL25_fda* labeled with a Spc^R^ gene and hosting IME_ *SsalL25_oriT* labeled with a Kan^R^ gene	[14] and this work
*S. salivarius*	L45(ICE_*SsalL45_rpsI* ::IME_*SsalL45*_*oriT*)	Wild type strain carrying ICE_*SsalL45_rpsI* hosting IME_*SsalL45*_*oriT*	[14]
*S. salivarius*	F1-4(ICE_*SsaF1-4_fda* Δ*int*)	Strain carrying ICE_*SsaF1-4_fda* with its integrase gene replaced by a Cm^R^ gene	[21] and this work
*Streptococcus thermophilus*	LMG18311(ICE*St3*)	Strain carrying ICE*St3* integrated in *fda* and labeled with a Cm^R^ or Spc^R^ gene	[22]
*S. thermophilus*	LMG18311(ICE*St3*Δ*int*)	Strain carrying ICE*St3* integrated in *fda* and labeled with a Spc^R^ gene and with its integrase gene replaced by a Cm^R^ gene	This work
*S. thermophilus*	LMG18311(ICE*St3*Δ*rel*)	Strain carrying ICE*St3* integrated in *fda* and labeled with a Cm^R^ gene and deleted for its relaxase gene	[23]
*S. thermophilus*	LMG18311(ICE*St3* ::IME_*SsalL25_oriT*)	Strain carrying ICE*St3* integrated in *fda* and labeled with a Cm^R^ gene and hosting IME_*SsalL25*_*oriT* Kan^R^	This work
*S. thermophilus*	LMG18311(ICE*St3* ::IME_*SsalL25_oriT*Δ*rel*)	Strain carrying ICE*St3* integrated in *fda* and labeled with a Cm^R^ gene and hosting IME_*SsalL25*_*oriT* Kan^R^ deleted for its relaxase gene	This work
*S. thermophilus*	LMG18311(ICE*St3* Δ*int*::IME_*SsalL25_oriT*)	Strain carrying ICE*St3* integrated in *fda* and Spc^R^ with its integrase gene replaced by a Cm^R^ gene and hosting IME_*SsalL25*_*oriT* Kan^R^	This work
*S. thermophilus*	LMG18311(ICE*St3* Δ*rel*::IME_*SsalL25_oriT*)	Strain carrying ICE_*St3* integrated in *fda* and Cm^R^ deleted for its relaxase gene and hosting IME_*SsalL25*_*oriT* Kan^R^	This work
*S. thermophilus*	LMG18311 Δ*epsE*	Strain deficient in exopolysaccharide production, Ery^R^	[24]
*S. thermophilus*	LMG18311 Δ*epsE*(ICE*St3*Δ*int*)	Strain deficient in exopolysaccharide production, Ery^R^ and carrying ICE*St3* Spc^R^ with its integrase gene replaced by a Cm^R^ gene	This work

^1^ As described in the material and methods, Integrative Conjugative Elements (ICEs) and Integrative Mobilizable Elements (IMEs) are named according to the bacterial species and strain in which they were identified and their site of insertion. Deletion of genes introduced into the element are also mentioned (*int* for integrase, *rel* for relaxase, and *espE* for exopolysaccharides production locus). For more clarity, antibiotic resistance genes used to label the elements are not included in the element name but are indicated in the genotype column; Spc^R^, spectinomycin resistance; Kan^R^, kanamycin resistance; Cm^R^, chloramphenicol resistance; Ery^R^, erythromycin resistance.

## Supplementary Materials

The following are available online at https://www.mdpi.com/2073-4425/11/9/1004/s1, Figure S1: Phylogenetic tree of integrases of the identified IME_*oriT*, Figure S2: Excision of IME_*SsalL25_oriT* and its hosting ICE*St3* in LMG18311, Table S1: Primers used in this study [43], Table S2: Characteristics of the IME_*oriT* identified in this work, Table S3: Summary of mating experiments.
