# Supplementary material for: Mobilization of IMEs Integrated in the oriT of ICEs Involves Their Own Relaxase Belonging to the Rep-Trans Family of Proteins
Source: Genes (Basel). 2020 Aug 26;11(9):1004. doi: 10.3390/genes11091004 (PMC7563843; doi:10.3390/genes11091004)
Supplement: Supplementary file 1 [file genes-11-01004-s001.zip › Figure S1 Phylogenetic tree integrases.pdf]

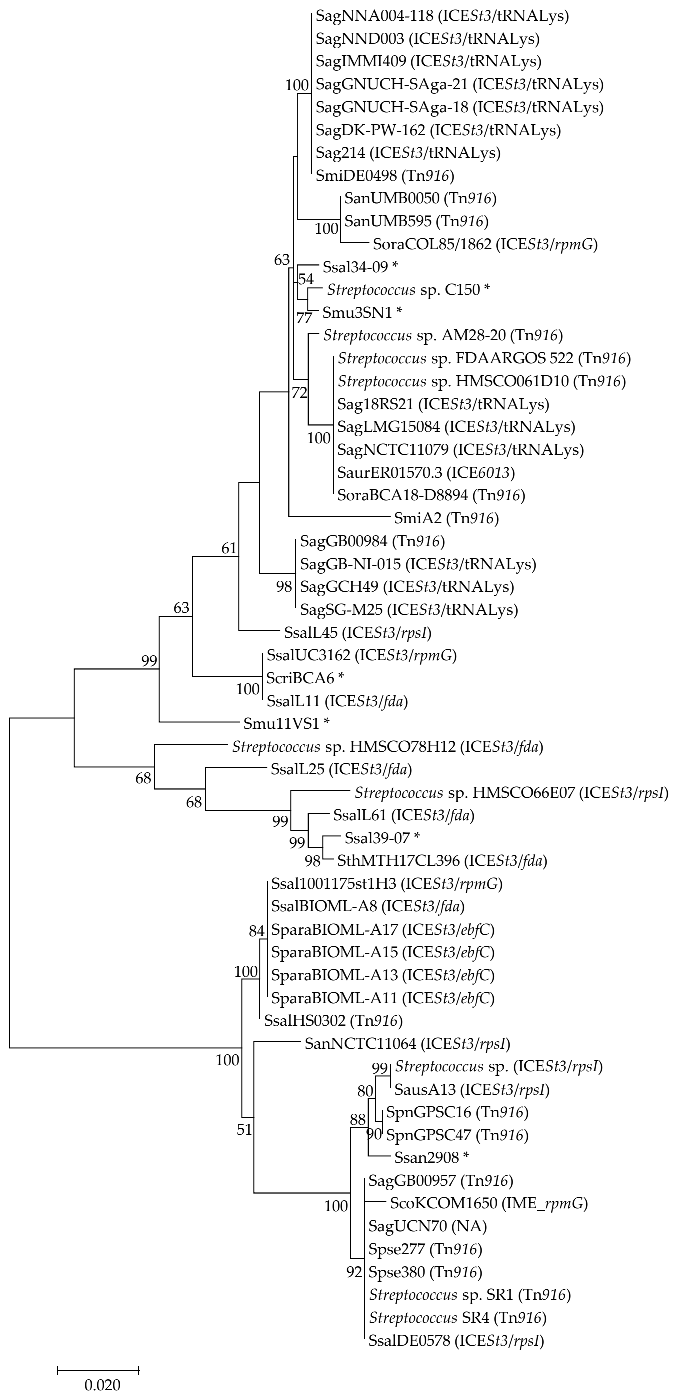

Figure S1. Molecular phylogenetic analysis of the sequences of integrase genes of IMEs detected in this work.

The integration site of the IME is indicated in brackets after the name of the strain: in an ICE (with indication of the family of the ICE and its integration site), in an IME or * if not in an ICE/IME.

The evolutionary history was inferred by using the Maximum Likelihood method based on the Tamura-Nei model [20]. The tree with the highest log likelihood is shown. The percentage of trees in which the associated taxa clustered together is shown next to the branches only when higher than 50%. Initial tree(s) for the heuristic search were obtained automatically by applying Neighbor-Join and BioNJ algorithms to a matrix of pairwise distances estimated using the Maximum Composite Likelihood approach, and then selecting the topology with superior log likelihood value. The tree is drawn to scale, with branch lengths measured in the number of substitutions per site. Evolutionary analyses were conducted in MEGA7 [19].
