# Supplementary material for: Mobilization of IMEs Integrated in the oriT of ICEs Involves Their Own Relaxase Belonging to the Rep-Trans Family of Proteins
Source: Genes (Basel). 2020 Aug 26;11(9):1004. doi: 10.3390/genes11091004 (PMC7563843; doi:10.3390/genes11091004)
Supplement: Supplementary file 1 [file genes-11-01004-s001.zip › Figure S2.pdf]

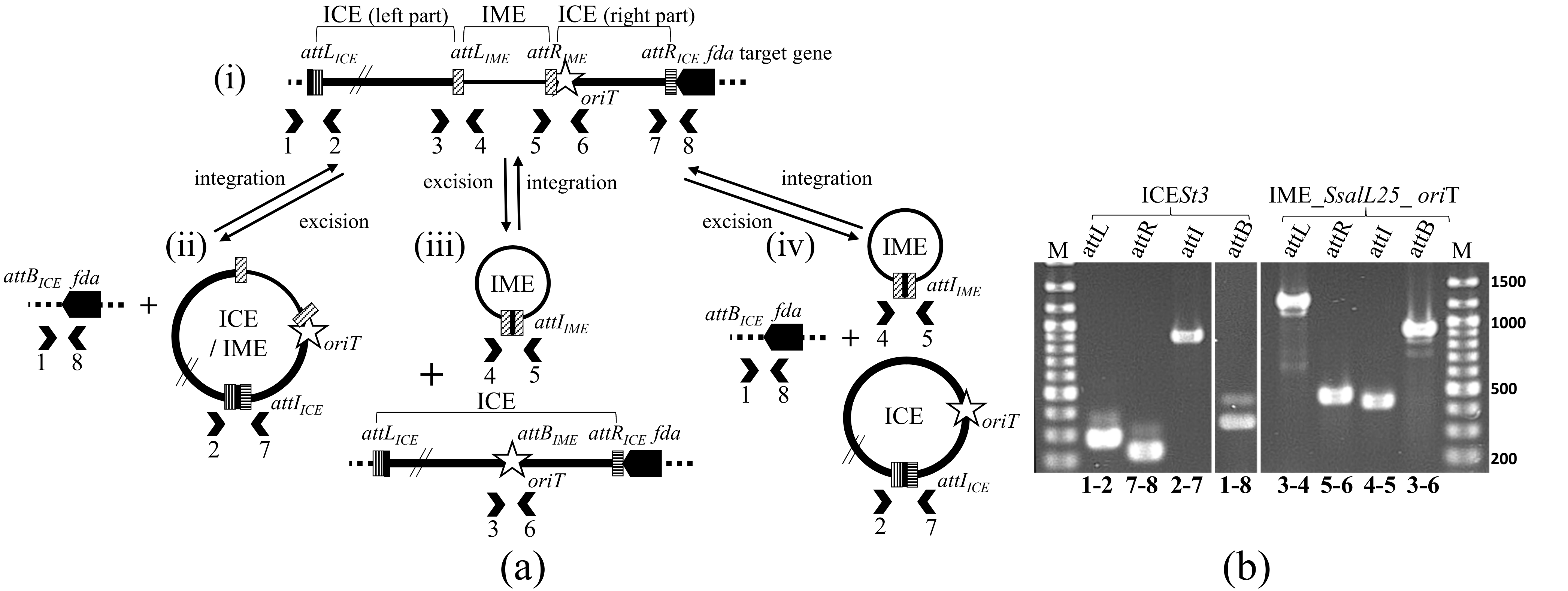

Figure S2: Excision of IME_*SsalL25_oriT* and its hosting ICE*St3* in *S. thermophilus* LMG18311. (**a**) Diagram showing the localization of PCR primers (numbered chevrons) used to detect (i) integrated forms of elements (*attR* and *attL* for right and left junctions, respectively) and excised forms (*attI* for circular form of the integrative elements and *attB* for the bacterial empty site) if (ii) the ICE/IME composite element excises, (iii) only the IME excises or (iv) both elements excise separately; (**b**) PCR products obtained with the primer pairs (numbers indicated at the bottom of the gel) when analyzing excision of IME_*SsalL25_oriT* and of its hosting ICE (ICE_*SsalL25_fda*). The sizes of the PCR fragments were confirmed by parallel migration of a DNA ladder (M for marker on the figure). The primers pairs used for these amplifications and expected sequence lengths are listed in Table S1.
