# Supplementary material for: Mobilization of IMEs Integrated in the oriT of ICEs Involves Their Own Relaxase Belonging to the Rep-Trans Family of Proteins
Source: Genes (Basel). 2020 Aug 26;11(9):1004. doi: 10.3390/genes11091004 (PMC7563843; doi:10.3390/genes11091004)
Supplement: Supplementary file 1 [file genes-11-01004-s001.zip › Figure S3 Phylogenetic tree relaxases.pdf]

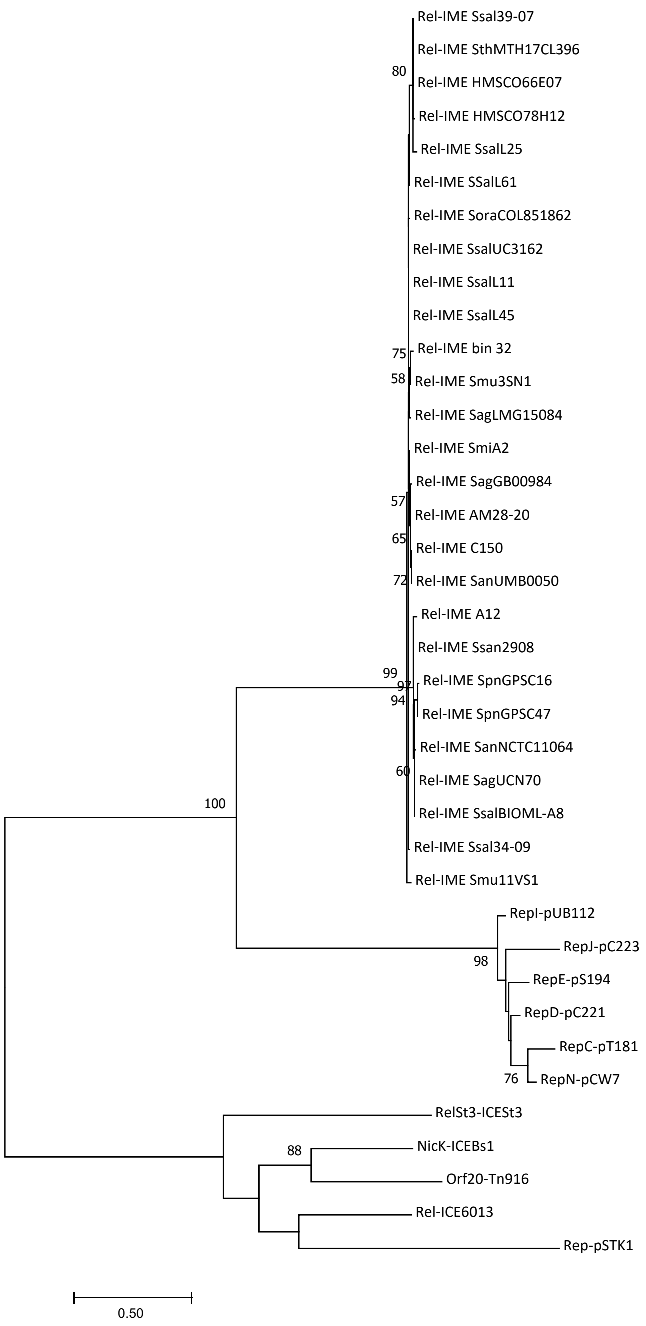

Figure S3. Molecular phylogenetic analysis of the sequences of relaxases of IMEs of the IME_*oriT* family, ICEs of the Tn*916* superfamily and Rep-trans proteins encoded by plasmids of Firmicutes.

Amino acid sequences were aligned using ClustalW. Sequences that were identical were included only once (see Table S2 for further details). The evolutionary history was inferred by using the Maximum Likelihood method based on the JTT matrix-based model. The tree with the highest log likelihood is shown. The percentage of trees in which the associated taxa clustered together is shown next to the branches only when higher than 50%. Initial tree(s) for the heuristic search were obtained automatically by applying Neighbor-Join and BioNJ algorithms to a matrix of pairwise distances estimated using a JTT model, and then selecting the topology with superior log likelihood value. The tree is drawn to scale, with branch lengths measured in the number of substitutions per site. Evolutionary analyses were conducted in MEGA7 [19].
