# Supplementary material for: Mobilization of IMEs Integrated in the oriT of ICEs Involves Their Own Relaxase Belonging to the Rep-Trans Family of Proteins
Source: Genes (Basel). 2020 Aug 26;11(9):1004. doi: 10.3390/genes11091004 (PMC7563843; doi:10.3390/genes11091004)
Supplement: Supplementary file 1 [file genes-11-01004-s001.zip › Table S1 primers.pdf]

TABLE S1. Primers used in this work.

Primer use	Primer designation (number for figure 2)	Sequence (5′to 3′)	Product size (pb)	Reference
IME_*SsalL25_oriT* labelling				
	IMEoriT_1	CACCACTCAAACTGTCATAAA		This study
	IMEoriT_2km	aacaagatccagaaaggatcttgtttttctagaCCGAACCTGGATAGTATAATTTC		This study
	IMEoriT_3km	aatctgcctcctcatcctcttcatcctctctagaGTCTATCGCCCTCGCATTAG		This study
	IMEoriT_4	GGAGCTCAATTTCTTCGTTTC		This study
	aphaA3_F	GCGATCCTAGAGAGGATGAAGAGGATGAGGAGGCAG		This study
	aphaA3Term_R	GCGACTCTAGAAAAAACACGATCCTTTCTGGATCTTGTTTTTGCTTTTTA GACATCTAAATCTAGG		This study
ICE_*SsalL25_fda* labelling				
	L25_ICE_1	CAGGATTCGCACAATAAGATAG		This study
	L25_ICE_2Spc	gggaaatattcattctaattggCATCCCAGTTGTATGGACT		This study
	L25_ICE_3Spc	gtcacgttacgataacttcgGAATACTGCTTCAAAAGATAAG		This study
	L25_ICE_4	TGCACATATAGTTGGTTTAGG		This study
	Spec_F	CGAAGTTATCGTAACGTGAC		This study
	Spec_R	CCAATTAGAATGAATATTTCCC		This study
ICEs_*fda Δint* deletion and labelling				
	Delta_intICE_1	GTGGCTCTTCTCAATCACTAAA		This study
	Delta_intICE_2Cm	cgtgttattccaccaatacgCCATTATCCCACCTCTTTAAATCG		This study
	Delta_intICE_3Cm	gctatttatcctaagcagagcACCTATCCTTGATACTCTTACACA		This study
	Delta_intICE_4	TGATAATGGTTACGCAGTTGG		This study
	K7catQ5'	CGTATTGGTGGAATAACACG		This study
	K7catQ3'	GCTCTGCTTAGGATAAATAGC		This study
ICE*St3 Δint* detection				
attR	K7catQ5'	CGTATTGGTGGAATAACACG	1834	This study
	Delta_intICE_4	TGATAATGGTTACGCAGTTGG		This study
IME_*SsalL25_oriT* and flanking region for transformation				
	L25orfKF	GTTGTCCTTATGACCATTAT		This study
	L25orfJR	CACACCGTTGATGTATTC		This study
IME_*SsalL25_oriT Δrel* deletion				

	Delta_relIME_1	**GGGCGGCCGC**TAACAATCTCCTCAAGTC		This study
	Delta_relIME _2	GATACTATCTTCATCAATCAT		This study
	Delta_relIME _3	atgattgatgaagatagtatcGAATTCCTAGAATTTACTAGT		This study
	Delta_relIME _4	**CGGGGCCC**AATCTGGTCTCTAATACTG		This study
Detection of ICEs and IMEs integrated and excised forms				
ICE_*SsalL11_fda*				
attL	attBfdaSsal_F	GCCCAACCAAATAACACTAAA	305	[43]
	attISt3_R	CGGTGTAATGGGAAGTATGG		[30]
attR	intICESt3-fda_F	ACCTATACTTGATACTCTTACACA	951	This study
	L25_intICE4	TGATAATGGTTACGCAGTTGG		This study
attI	intICESt3-fda_F	ACCTATACTTGATACTCTTACACA	153	This study
	attISt3_R	CGGTGTAATGGGAAGTATGG		[30]
attB	attBfdaSsal_F	GCCCAACCAAATAACACTAAA	1084	[43]
	L25_intICE4	TGATAATGGTTACGCAGTTGG		This study
IME_*SsalL11_oriT*				
attL	CP ICE Ssa2_F	GTCTTTATCATCTTTGCCAT	1206	This study
	L11-IMEcds32_R	AACAATTATCCGATTTGGAAG		This study
attR	intIME_oriT_F	CTGCTCTTTATTGGGAAG	759	This study
	relICE_R	GGCCTGTTTGTTTTCTGA		This study
attI	L11-IMEcds32_R	AACAATTATCCGATTTGGAAG	1097	This study
	intIME_oriT_F	CTGCTCTTTATTGGGAAG		This study
attB	CP ICE Ssa2_F	GTCTTTATCATCTTTGCCAT	890	This study
	relICE_R	GGCCTGTTTGTTTTCTGA		This study
ICE_*SsalL25_fda*				
attL	attBfdaSsal_F (1)	GCCCAACCAAATAACACTAAA	442	[43]
	attISt3_R (2)	CGGTGTAATGGGAAGTATGG		[30]
attR	intICESt3-fda_F (7)	ACCTATACTTGATACTCTTACACA	951	This study
	L25_intICE4 (8)	TGATAATGGTTACGCAGTTGG		This study
attI	intICESt3-F (7)	AGGGCTTTCTGACGAATTAG	891	This study
	attISt3_R (2)	CGGTGTAATGGGAAGTATGG		[30]
attB	attBfdaSsal_F (1)	GCCCAACCAAATAACACTAAA	1206	[43]
	L25_intICE4 (8)	TGATAATGGTTACGCAGTTGG		This study
var_L25	L25varICE_F	CGAAAGGAGATCAGTATTAG	724	This study

	L25varICER	GGCTAGAACCAGAAATAAA		This study
IME_*SsalL25_oriT*				
attL	CP ICE Ssa2_F (3)	GTCTTTATCATCTTTGCCAT	1277	This study
	L25-IMEcds20_R (4)	GTATTCAATGTTCCGCTATG		This study
attR	IME_oriT_F (5)	TACATTCAACCAAAAGGT	487	This study
	relICE_R (6)	GGCCTGTTTGTTTTCTGA		This study
attI	IME_oriT_F (5)	TACATTCAACCAAAAGGT	417	This study
	IME_oriT_R (4)	TGGTAGCTTTTTGGTAGC		This study
attB	relICE_R (6)	GGCCTGTTTGTTTTCTGA	921	This study
	CP ICE Ssa2_F (3)	GTCTTTATCATCTTTGCCAT		This study
ICE_*SsalL45_rpsI*				
attL	N20cds18_F	CCAAGCCGTCGTCATAATAG	1173	[43]
	JIMcds19_F	TTCTGGTTGGGATCGTTATG		This study
attR	IntRpsI_F1	TAWTCWGARGCHYTWACKCATAG	297	This study
	RpsI_F	CAGACCTTCGTCTCGTTATC		This study
attI	IntRpsI_F1	TAWTCWGARGCHYTWACKCATAG	331	This study
	JIMcds19_F	TTCTGGTTGGGATCGTTATG		This study
attB	N20cds18_F	CCAAGCCGTCGTCATAATAG	1139	[43]
	RpsI_F	CAGACCTTCGTCTCGTTATC		This study
IME_*SsalL45_oriT*				
attL	CP ICE Ssa2_F	GTCTTTATCATCTTTGCCAT	1014	This study
	L45-IMEcds40_R	GCAATCAATACTCCAACAAG		This study
attR	intIME_oriT_F	CTGCTCTTTATTGGGAAG	759	This study
	relICE_R	GGCCTGTTTGTTTTCTGA		This study
attI	intIME_oriT_F	CTGCTCTTTATTGGGAAG	902	This study
	L45-IMEcds40_R	GCAATCAATACTCCAACAAG		This study
attB	CP ICE Ssa2_F	GTCTTTATCATCTTTGCCAT	899	This study
	relICE_R	GGCCTGTTTGTTTTCTGA		This study
ICE_*SsalF1-4_fda*				
attR	catQ1	TAGAAAGCCATACTTTGAGC	1883	This study
	L25_intICE4	TGATAATGGTTACGCAGTTGG		This study
attI	catQ1	TAGAAAGCCATACTTTGAGC	1103	This study
	attISt3_R	CGGTGTAATGGGAAGTATGG		This study

ICE*St3*				
attL	132.3	GGACTACTAAGAGAACAT	273	[22]
	attISt3_R	CGGTGTAATGGGAAGTATGG		This study
attR	intICESt3-fda_F	ACCTATACTTGATACTCTTACACA	227	This study
	attBSt3_R	CTCTTCGACCCACGTAAATTC		This study
attI	attISt3_R	CGGTGTAATGGGAAGTATGG	887	This study
	attBSt3_R	CTCTTCGACCCACGTAAATTC		This study
attB	132.3	GGACTACTAAGAGAACAT	361	[22]
	131.2	TGTTGCTGAATACGAAGC		[22]
ermB in *ΔepsE S. thermophilus* strains				
	EG940	TGTAATTAAGAAGGAGTGA	766	[24]
	EG941	TAGAATTATTTCCTCCCGT		[24]

The primer sequences are capitalized when the sequence is complementary to the amplified region. Lower case letters are complementary to a primer allowing the amplified region to overlap. Bold letters indicate the restriction sites *Apa*I and *Not*I (underlined) and extra nucleotides to ensure optimal digestion of PCR amplified region by restriction enzymes prior to cloning in plasmid pG+host9 [23].
