# Supplementary material for: Mobilization of IMEs Integrated in the oriT of ICEs Involves Their Own Relaxase Belonging to the Rep-Trans Family of Proteins
Source: Genes (Basel). 2020 Aug 26;11(9):1004. doi: 10.3390/genes11091004 (PMC7563843; doi:10.3390/genes11091004)
Supplement: Supplementary file 1 [file genes-11-01004-s001.zip › Table S3.pdf]

Table S3: Summary of mating experiments. Transfer frequencies (of ICE or IME) are indicated as transconjugant per donor cell. The detection level for ICE*St3* and its mutants and IME_*SsalL25_oriT* and its mutants are 10^{-6} and 10^{-7} respectively so when the frequency was below this detection level, ND ("not detected") is indicated. If the donor strain does not carry the IME_*SsalL25_oriT* or if ICE transconjugants cannot be identified due to the presence of a resident ICE in the recipient strain, it has been indicated in the table as "**-**". Mating pairs not tested were left empty.

	**Recipient strains**			
	LMG18311 Δ*epsE*		LMG18311 Δ*epsE* (ICE*St3*Δ*int*)	
**Donor strains**	ICE	IME	ICE	IME
LMG18311(ICE*St3*)	$2.3 \times 10^{-4} \pm 0.8 \times 10^{-4}$ *	-		
LMG18311(ICE*St3*::IME_*SsalL25_oriT*)	$3.3 \times 10^{-4} \pm 1.3 \times 10^{-4}$ *	$3.1 \times 10^{-6} \pm 1.5 \times 10^{-6}$ *	-	$9.3 \times 10^{-6} \pm 6.5 \times 10^{-6}$ *
LMG18311(ICE*St3*Δ*int*)	ND	-		
LMG18311(ICE*St3*Δ*int*::IME_*SsalL25_oriT*)	ND	ND	-	$4.8 \times 10^{-6} \pm 4.0 \times 10^{-6}$ *
LMG18311(ICE*St3*Δ*rel*)	ND	-		
LMG18311(ICE*St3*Δ*rel*::IME_*SsalL25_oriT*)	ND	ND		
LMG18311(ICE*St3*::IME_*SsalL25_oriT*Δ*rel*)	$3.5 \times 10^{-4} \pm 2.0 \times 10^{-4}$ *	ND	-	ND

* transconjugants per donor cell
